# Influence of Intermittent Hypoxia/Hypercapnia on Atherosclerosis, Gut Microbiome, and Metabolome

**DOI:** 10.3389/fphys.2021.663950

**Published:** 2021-04-08

**Authors:** Jin Xue, Celeste Allaband, Dan Zhou, Orit Poulsen, Cameron Martino, Lingjing Jiang, Anupriya Tripathi, Emmanuel Elijah, Pieter C. Dorrestein, Rob Knight, Amir Zarrinpar, Gabriel G. Haddad

**Affiliations:** ^1^Department of Pediatrics, University of California, San Diego, San Diego, CA, United States; ^2^Biomedical Sciences Program, University of California, San Diego, San Diego, CA, United States; ^3^Division of Gastroenterology, University of California, San Diego, San Diego, CA, United States; ^4^Bioinformatics and Systems Biology Program, University of California, San Diego, San Diego, CA, United States; ^5^Center for Microbiome Innovation, University of California, San Diego, San Diego, CA, United States; ^6^Division of Biostatistics, University of California, San Diego, San Diego, CA, United States; ^7^Division of Biological Sciences, University of California, San Diego, San Diego, CA, United States; ^8^Skaggs School of Pharmacy and Pharmaceutical Sciences, University of California, San Diego, San Diego, CA, United States; ^9^Collaborative Mass Spectrometry Innovation Center, University of California, San Diego, San Diego, CA, United States; ^10^Department of Computer Science and Engineering, University of California, San Diego, San Diego, CA, United States; ^11^Division of Gastroenterology, VA San Diego, La Jolla, CA, United States; ^12^Institute of Diabetes and Metabolic Health, University of California, San Diego, San Diego, CA, United States; ^13^Department of Neuroscience, University of California, San Diego, San Diego, CA, United States; ^14^Rady Children’s Hospital, San Diego, CA, United States

**Keywords:** obstructive sleep apnea, atherosclerosis, intermittent hypoxia and hypercapnia, microbiome, metabolome

## Abstract

Obstructive sleep apnea (OSA), a common sleep disorder characterized by intermittent hypoxia and hypercapnia (IHC), increases atherosclerosis risk. However, the contribution of intermittent hypoxia (IH) or intermittent hypercapnia (IC) in promoting atherosclerosis remains unclear. Since gut microbiota and metabolites have been implicated in atherosclerosis, we examined whether IH or IC alters the microbiome and metabolome to induce a pro-atherosclerotic state. Apolipoprotein E deficient mice (*ApoE^−/−^*), treated with IH or IC on a high-fat diet (HFD) for 10 weeks, were compared to Air controls. Atherosclerotic lesions were examined, gut microbiome was profiled using 16S rRNA gene amplicon sequencing and metabolome was assessed by untargeted mass spectrometry. In the aorta, IC-induced atherosclerosis was significantly greater than IH and Air controls (aorta, IC 11.1 ± 0.7% vs. IH 7.6 ± 0.4%, *p* < 0.05 vs. Air 8.1 ± 0.8%, *p* < 0.05). In the pulmonary artery (PA), however, IH, IC, and Air were significantly different from each other in atherosclerotic formation with the largest lesion observed under IH (PA, IH 40.9 ± 2.0% vs. IC 20.1 ± 2.6% vs. Air 12.2 ± 1.5%, *p* < 0.05). The most differentially abundant microbial families (*p* < 0.001) were Peptostreptococcaceae, Ruminococcaceae, and Erysipelotrichaceae. The most differentially abundant metabolites (*p* < 0.001) were tauro-β-muricholic acid, ursodeoxycholic acid, and lysophosphoethanolamine (18:0). We conclude that IH and IC (a) modulate atherosclerosis progression differently in distinct vascular beds with IC, unlike IH, facilitating atherosclerosis in both aorta and PA and (b) promote an atherosclerotic luminal gut environment that is more evident in IH than IC. We speculate that the resulting changes in the gut metabolome and microbiome interact differently with distinct vascular beds.

## Introduction

Obstructive sleep apnea (OSA) is a common disorder characterized by repetitive episodes of complete or partial upper airway obstruction during sleep. These apneic episodes lead to intermittent hypoxia and hypercapnia (IHC), wide intrathoracic pressure swings, as well as sleep fragmentation. OSA affects approximately 9–38% of the general adult population with 13–33% in men and 6–19% in women (Senaratna et al., 2017). Advanced age, male gender, and higher body-mass index increase OSA prevalence (Senaratna et al., 2017). OSA is independently associated with the elevated risk of myocardial infarction, stroke, and cardiovascular mortality, mainly through the promotion of severe atherosclerosis (Drager et al., 2011; Lui and Sau-Man, 2012; Sarkar et al., 2018; Tietjens et al., 2019). The pathophysiological mechanisms underlying OSA associated atherosclerotic risk are not completely understood.

Chronic intermittent hypoxia (IH), generated during recurrent apneic episodes, is a major factor linking OSA to cardiovascular diseases including atherosclerosis (Drager et al., 2011). Intermittent hypercapnia (IC) also occurs in OSA but is usually not evaluated in most OSA-based translational studies. IC can potentially affect the formation of atheroma (Xue et al., 2017) as well. In the present investigation, we examined the lesion formation under these different study conditions (i.e., IH and IC) to better define the role of each component in atherosclerosis.

Considerable evidence indicates that human gut microbiota contributes to cardiovascular diseases, including atherosclerosis and metabolic disorders, e.g., obesity and type 2 diabetes, both of which are atherogenic (Jie et al., 2017; Jonsson and Backhed, 2017; Tang et al., 2017). Gut microbiota likely affects atherosclerosis through several mechanisms: (1) bacterial infection activates the immune system and triggers a harmful inflammatory response that aggravates plaque progression and rupture, (2) cholesterol and lipid metabolism altered by gut microbiota affect the development of atherosclerosis, and (3) microbial metabolites have either beneficial or deleterious effects on atherosclerosis (Jonsson and Backhed, 2017; Komaroff, 2018). Our recent studies indicate that the latter of these three likely plays an important role in OSA-induced atherosclerosis (Xue et al., 2017; Tripathi et al., 2018). Although trimethyalamine-N-oxide is perhaps the most well-known example of an atherogenic bacterial metabolite, bile acids and phosphocholines could be intermediates or involved in atherogenesis. For example, tauro-β-muricholic acid (TβMCA) is an FXR antagonist (Sayin et al., 2013) and can contribute to atherosclerosis (Hanniman et al., 2005; Ding et al., 2018). Ursodeoxycholic acid (UDCA) has been demonstrated to have anti-inflammatory effects, which could alleviate the development of atherosclerosis (Ko et al., 2017). Lysophospholipids have also been implicated deleterious roles in atherosclerosis (Matsumoto et al., 2007; Li et al., 2016b). Interestingly, gut microbiota composition is altered in different mouse models of OSA (Moreno-Indias et al., 2015; Xue et al., 2017; Tripathi et al., 2018). In this work, we explored the changes in gut microbiota population and metabolites induced by IH and IC separately. The knowledge obtained will help us to dissect out the individual impact of IH and IC on gut microbiota and metabolites as well as on atherosclerosis formation.

We hypothesized that IH or IC induces specific alterations in the gut microbiome and their metabolites, which may promote atherosclerosis. The questions we sought to address in the current study were (1) what is the particular role of IH or IC in inducing or promoting atherosclerosis, (2) what is the response of the vascular system (i.e., aorta vs. pulmonary artery, PA) to IH or IC in term of atherogenesis, (3) what is the signature of IH or IC on gut microbiome and metabolites, and (4) what is the potential impact of these changes of gut microbiome and metabolites on the development of atherosclerosis?

## Materials and Methods

### Animals With High-Fat Diet

Atherosclerosis-prone 10-week old male *ApoE*^−/−^ mice on C57BL/6 J background (002052; The Jackson Laboratory, Bar Harbor, ME) were used (Piedrahita et al., 1992), and *ApoE* deficiencies were confirmed by PCR. The mice were given a high-fat diet (HFD) containing 1.25% cholesterol and 21% milk fat (4.5 Kcal/g; TD.96121; Envigo-Teklad Madison, WI) for 10 weeks while being exposed to either IH, IC, or room air. All animal protocols were approved by the Animal Care Committee of the University of California, San Diego and followed the Guide for the Care and Use of Laboratory Animals of the National Institutes of Health.

### Intermittent Hypoxia and Hypercapnia Exposure

Intermittent hypoxia (IH) or intermittent hypercapnia (IC) was administered in a computer-controlled atmosphere chamber (OxyCycler, Reming Bioinstruments, Redfield, NY) as previously described (Xue et al., 2017). Mice were exposed to 8% O_2_ or 8% CO_2_ for short periods (∼4 min) separated by alternating periods (∼4 min) of normoxia [(O_2_) = 21%] and normocapnia [(CO_2_) = ∼0.5%)] with 1–2 min ramp intervals, 10 min per cycle, 10 h per day during the light cycle, for 10 weeks. Control mice were on the same HFD but in room air (21% O_2_ and 0.5% CO_2_).

### Quantification of Atherosclerotic Lesions

Atherosclerosis was quantified by computer-assisted image analysis (ImageJ, NIH Image; Schneider et al., 2012) in Sudan Red-stained en face preparations of the aorta and pulmonary arteries as previously described (Xue et al., 2017). The extent of lesion was presented by the percentage of Sudan IV-stained area to the total area of the tissue examined. Images of the aortic arch were cropped from the rest of the aorta by measuring the same distance from the bifurcation to the aortic body using photo-editing software (Adobe Photoshop CS6, Adobe Systems Inc., San Jose, CA). All the measurements were done by blinded investigators. Data were presented as means ± SEM. One-way ANOVA with Tukey’s multiple comparison test was employed and *p* < 0.05 was considered as statistically significant.

### Microbiome

Fecal samples were collected consistently between 9 AM and 11 AM (ZT3-ZT5) on collection days and stored at −80°C until the end of the study. We chose to collect samples at ZT3-ZT5 due to a concomitant circadian study from our group, indicating that it was the time of greatest microbiome composition differences between IHC and Air (Unpublished data). Then, samples were prepared for sequencing and analysis in a manner consistent with the Earth Microbiome Project standard protocols (Caporaso et al., 2012).^1^ The V4 region of 16S rRNA gene was sequenced using the primer pair 515f to 806r with Golay error-correcting barcodes on the reverse primer. After processing, raw sequence data were uploaded to Qiita (Gonzalez et al., 2018; QIITA #11829) and were processed using the Deblur (Amir et al., 2017) workflow with default parameters into a BIOM format table. The BIOM table was processed through QIIME 2 (version 2019.10; Bolyen et al., 2019). Dataset were rarified to 9,400 reads to control for sequencing effort. Weighted UniFrac (Chang et al., 2011) distances were used for microbiome PCoA plots and significance was tested using PERMANOVA (Anderson, 2017). The assigned sequence variants (ASVs) were collapsed to the Family taxonomic level. Differential abundance screening was performed using a permutation-based test with FDR correction in Calour (Jiang et al., 2017; Xu et al., 2019).^2^ The bacterial families were selected based on significance present under IC and IH conditions as well as previously known influence on phenotype. Data were visualized using EMPeror (Vazquez-Baeza et al., 2013) and custom python scripts.^3^

### Metabolome

Each fecal sample was examined by untargeted liquid chromatography-tandem mass spectrometry (LC-MS/MS) in the same manner as described previously (Tripathi et al., 2018). In brief, the samples were homogenized, transferred to a standard 96-well plate, and analyzed on a Vanquish ultrahigh-performance liquid chromatography (UPLC) system coupled to a Q Exactive orbital ion trap (Thermo Fisher Scientific, Bremen, Germany). A C18 core shell column (Kinetex column, 50 by 2 mm, 1.7-μm particle size, 100-Å pore size; Phenomenex, Torrance, CA) was used for chromatographic separation. Raw spectra were converted to *m/z* extensible markup language (mzXML) in centroid mode using MSConvert (part of ProteoWizard; Chambers et al., 2012). After isotope peak removal and alignment of peaks, MZmine2 (Pluskal et al., 2010) was used to create a feature matrix containing the feature retention times, and the exact mass and peak areas of the corresponding extracted ion chromatograms. Identification of molecular features was performed using MS1-based feature detection and MS2-based molecular networking using the GNPS workflow.^4^ The actual GNPS jobs can be found at the following URL: https://gnps.ucsd.edu/ProteoSAFe/status.jsp?task=78acff728c48421497ebf59441e18ea4. We used authentic bile acid standards from Cayman Chemical (Ann Arbor, MI) for level 1 identification of metabolites as defined by the 2007 metabolomics standards initiative (Sumner et al., 2007). There was an annotation rate of 41% (556/1359). The unannotated frequency table was analyzed using QIIME2 (version 2019.10). Canberra distances (Lance and Williams, 1967) were used for metabolomic PCoA plots and significance was tested using PERMANOVA (Anderson, 2017). Data were visualized using EMPeror (Vazquez-Baeza et al., 2013). Differential abundance screening was performed using a permutation-based test with FDR correction in Calour (Jiang et al., 2017; Xu et al., 2019). Features were selected based on significance present under IC and IH conditions as well as previously known influence on phenotype. The MS/MS spectral annotations were determined by using MS/MS-based spectral library matches on GNPS for level 3 identification of all non-bile acid molecules represented (Sumner et al., 2007). Bile acid standards were run using the same method for level 1 identification of all represented bile acids. After initial processing, a single sample that clustered with blank controls was dropped from further analyses. Each data point was normalized to total absorbance (the raw absorbance value of each metabolite in a sample/total absorbance for that sample) to calculate the relative abundances. Values were then plotted together using custom python scripts.^5^

### Longitudinal Data Statistical Analysis

For both the microbiome and metabolome, linear mixed effect modeling was performed using q2-longitudinal (Bokulich et al., 2018) to determine if the highlighted feature was significantly different between exposure conditions (Air/IH/IC) with respect to time. The formula used was Feature (microbiome or metabolome) ~ host_age∗exposure_type.

### Data Availability

Microbiome: EBI accession ERP110592.

Metabolome: IH/IC MSV000082973, MassIVE link for IH/IC: https://massive.ucsd.edu/ProteoSAFe/dataset.jsp?task=2ee8ad1a1c764aaf96677d480617c040.

GitHub link: https://github.com/knightlab-analyses/longitudinal-osa.

## Results

### Different Impacts of IH and IC on the Progression of Atherosclerosis in the Aorta, Aortic Arch, and Pulmonary Artery

**Figure 1:**
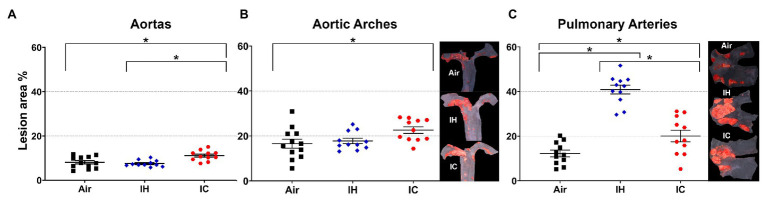
Atherosclerotic lesions in the **(A)** aortas, **(B)** aortic arches, and **(C)** pulmonary arteries of *ApoE*^−/−^ mice after 10-week treatments. Mice were exposed to either Air, IH, or IC for 10 weeks. The en-face lesions were quantified as the percentage of lesion area in the total area of the blood vessel examined. Side panels: representative Sudan IV-stained images of aortic arch **(B)** and PA **(C)**. In the aortas and aortic arches, *n* = 12 for Air, *n* = 11 for IH, and *n* = 11 for IC. In the pulmonary arteries, *n* = 11 for each Air, IH, and IC group. Data were presented as means ± SEM. Statistical significance (one-way ANOVA with Tukey’s multiple comparison test), *p < 0.05.

OSA patients suffer from oscillatory gas changes (IH and IC). To find out the effect of these gas changes on atherosclerosis, we examined the atherosclerotic lesions in the aorta, aortic arch, and PA after 10 weeks of IH or IC exposure in the presence of HFD. Our data showed that there were significant increases in lesion formation in the aorta and aortic arch in IC exposed mice as compared with Air controls (aorta, Air 8.1 ± 0.8% vs. IC 11.1 ± 0.7%, *p* < 0.05 and aortic arch, Air 16.6 ± 2.0% vs. IC 22.6 ± 1.5%, *p* < 0.05; Figures 1A,B). However, IH-treated mice showed a similar degree of lesions as Air controls without a significant difference (aorta, Air 8.1 ± 0.8% vs. IH 7.6 ± 0.4%, *p* > 0.05 and aortic arch, Air 16.6 ± 2.0% vs. IH 17.8 ± 1.2%, *p* > 0.05; Figures 1A,B). In the PA, the larger atherosclerotic formation was detected in the IH group, followed by IC, both of them showing significantly greater lesions than Air controls (PA, IH 40.9 ± 2.0% vs. IC 20.1 ± 2.6% vs. Air 12.2 ± 1.5%, *p* < 0.05; Figure 1C). Our data suggest that (1) IH promotes atherosclerosis much more in the PA than in the aorta and (2) IC contributes to atherosclerosis in the aorta, aortic arch, and PA.

### Different Impacts of IH and IC on the Gut Microbiome and Metabolome

Examination of 16S rRNA gene amplicon sequencing data of IH/IC/Air resulted in the selection of the top most differentially abundant microbial families (Supplementary Table S1). The ASVs were grouped together at the Family level for comparisons. Differential abundance testing was calculated by permutation test with discrete FDR correction in Calour (Xu et al., 2019). The top six differentially abundant families were Peptostreptococcaceae, Ruminococcaceae, Erysipelotrichaceae, Verrucomicrobiaceae, Coriobacteriaceae, and Lachnospiraceae. The significance of each selected family with respect to time and experimental condition was determined by linear mixed effect modeling.

Data from untargeted liquid chromatography with tandem mass spectrometry revealed 116 unique metabolites in common (Supplementary Table S1). Metabolites were divided into five different categories: phosphocholines, acylcarnitines, phosphoethanolamines, bile acids, and other. The other category included amino acids, dipeptides, and other small molecules. The following figures displayed six representative metabolites from each of the different categories based on differential abundance testing calculated by permutation test with discrete FDR correction (Xu et al., 2019), the greatest differences noted in at least one condition, and relevance to atherosclerosis. The six highlighted metabolites were TβMCA (bile acid), UDCA (bile acid), 1-stearoyl-2-hydroxy-sn-glycero-3-phosphoethanolamine [LysoPE(18:0); phosphoethanolamines], 1-hexadecyl-sn-glycero-3-phosphocholine (Lyso-PAF C-16; phosphocholines), oleoyl L-carnitine (acylcarnitines), and tryptophan (other).

#### IH Vs. Air

**Figure 2:**
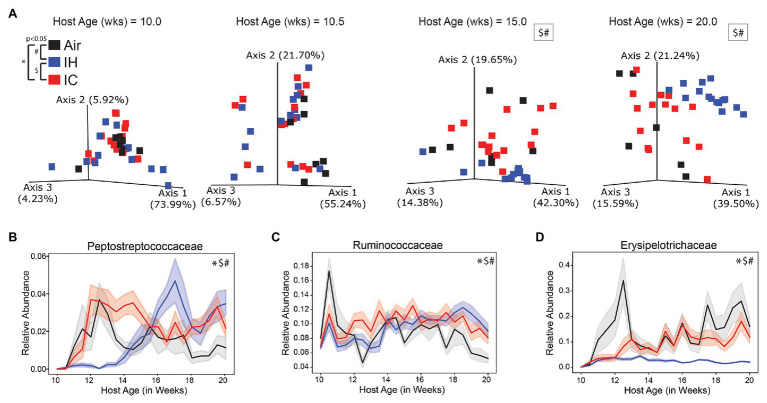
16S microbiome of *ApoE*^−/−^ mice on HFD during chronic 10-week treatment. **(A)** Weighted UniFrac PCoA of the microbiome during four key time points, each time point calculated in isolation. Longitudinal relative abundance values for **(B)** family Peptostreptococcaceae; **(C)** family Ruminococcaceae; and **(D)** family Erysipelotrichaceae. PERMANOVA used for statistical comparisons at the population level. Linear mixed effect (LME) modeling used for statistical comparisons of individual families over time. The shaded areas in parts **(B–D)** represent standard error of the mean. Air/controls are black (*n* = 6), IH is blue (*n* = 12), and IC is red (*n* = 12). Statistical significance *p* < 0.05, *IC vs. Air, ^#^IH vs. Air, and ^$^IH vs. IC.

**Figure 3:**
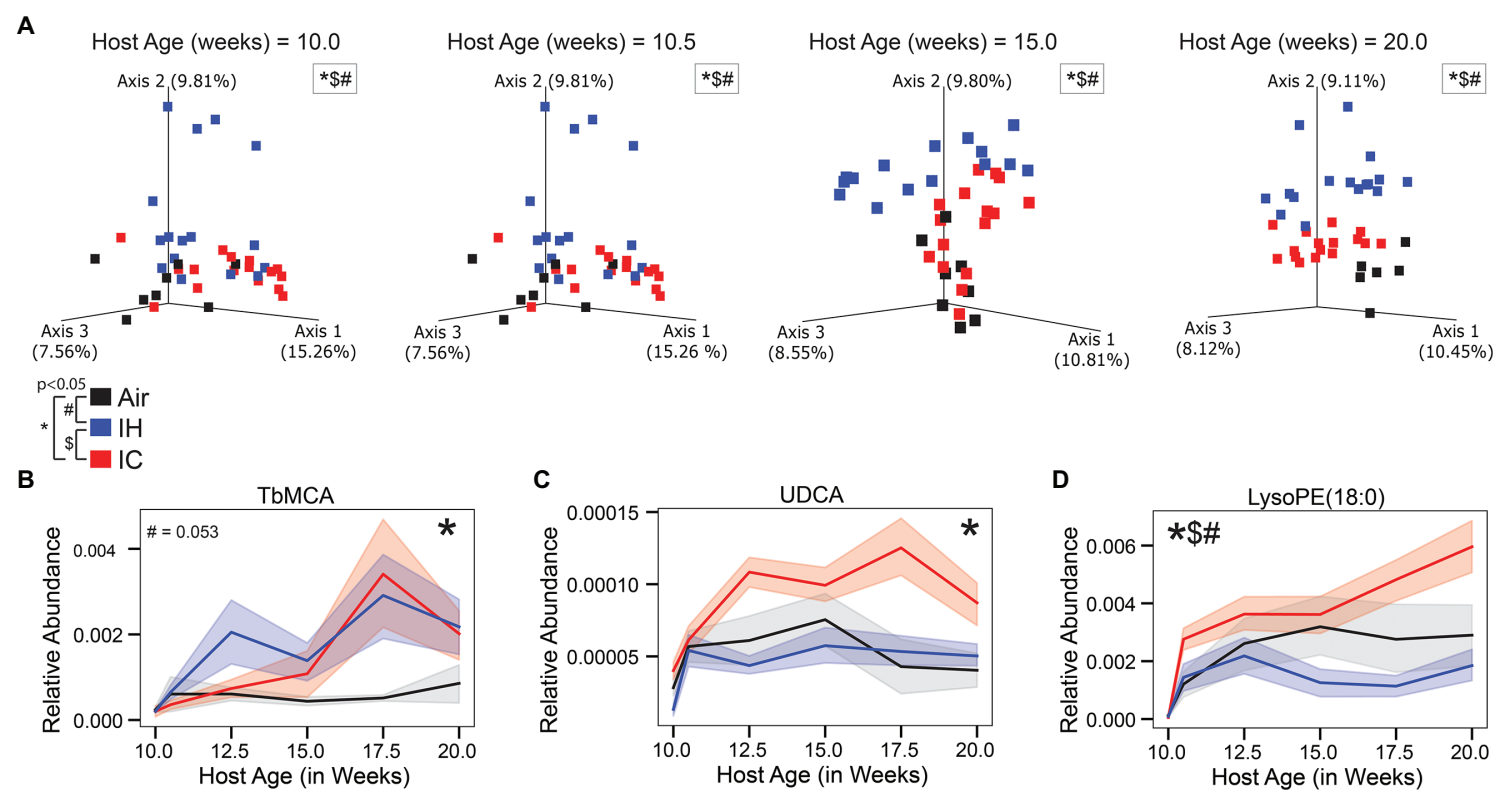
Untargeted LC-MS/MS metabolomics of *ApoE*^−/−^ mice on HFD during chronic 10-week treatment. **(A)** Canberra PCoA of the metabolome during four key time points, each time point calculated in isolation. Longitudinal relative abundance values for **(B)** Tauro-β-muricholic acid (TβMCA; level 1 identification); **(C)** Ursodeoxycholic acid (UDCA; level 1 identification); and **(D)** lysophosphoethanolamine 1-stearoyl-2-hydroxy-sn-glycero-3-phosphoethanolamine [LysoPE(18:0); level 3 identification]. PERMANOVA used for statistical comparisons at the population level. LME modeling used for statistical comparisons of individual metabolites over time. The shaded areas in parts **(B–D)** represent standard error of the mean. Air/controls are black (*n* = 6), IH is blue (*n* = 12), and IC is red (*n* = 12). Statistical significance *p* < 0.05, *IC vs. Air, ^#^IH vs. Air, and ^$^IH vs. IC.

Mice under IH conditions diverged significantly from Air controls over time in the beta diversity distances of both the microbiome (Figure 2A; Supplementary Figures S1A, S3A,C; pseudo-F 39.775; *p* < 0.001) and metabolome (Figure 3A; Supplementary Figures S2A, S3B,D; pseudo-F 5.963; *p* = 0.002). The microbiome had a lower value of *p* and higher pseudo-*F* value than the metabolome, indicating greater separation between the two conditions for the microbiome.

All the microbial families shown (Figures 2B–D and Supplementary Figures S1C–E) had significant differences between IH-conditioned mice and Air controls. Supplementary Figure S1B was a heatmap of the top six differential microbial families under Air, IH, and IC conditions and presented a global overview of the changes in gut microbiota over the treatment time. Pro-atherosclerotic Peptostreptococcaceae (Koeth et al., 2013; *p* < 0.001; Figure 2B) initially showed reduced relative abundance compared to controls. But at week 14, 2 weeks before the phenotype was known to appear, the relative abundance started to dramatically increase in comparison to controls. The reason for the switch was unclear. Interestingly, anti-atherosclerotic Verrucomicrobiaceae (Cani and de Vos, 2017; *p* < 0.001; Supplementary Figure S1D) exhibited the opposite trend change to Peptostreptococcaceae, i.e., relative abundance was higher during the first 4 weeks of IH exposure then became significantly lower than controls afterward. IH-conditioned mice had increased relative abundances in pro-atherosclerotic Ruminococcaceae (Wang et al., 2015; Liu et al., 2018; *p* < 0.001; Figure 2C), pro-atherosclerotic Coriobacteriaceae (Karlsson et al., 2012; *p* < 0.001; Supplementary Figure S1C), and pro-atherosclerotic Lachnospiraceae (Wang et al., 2015; Liu et al., 2018; *p* < 0.001; Supplementary Figure S1E) compared to Air control mice over the course of the experiment. Conversely, IH-conditioned mice had decreased relative abundances of anti-atherosclerotic Erysipelotrichaceae (Qiu et al., 2018; *p* = 0.009; Figure 2D).

Three of the six represented metabolites were significantly different between IH-conditioned mice and controls over the course of the study. Supplementary Figure S2B was a heatmap of six differential metabolites under Air, IH, and IC conditions and presented a global overview of their changes over the treatment time. Compared to control mice, IH-conditioned mice had decreased relative abundance of LysoPE (18:0; *p* = 0.031; Figure 3D) and increased relative abundances of pro-inflammatory Lyso-PAF C-16 (*p* < 0.001; Supplementary Figure S2C) and oleoyl L-carnitine (*p* = 0.002; Supplementary Figure S2D). Of note, the relative abundance of pro-atherosclerotic bile acid TβMCA was increased, and relative abundances of anti-atherogenic bile acid UDCA and anti-inflammatory tryptophan were decreased in IH-conditioned mice *via* permutation test with discrete FDR correction (TβMCA, *p* < 0.001; UDCA, *p* < 0.001; and tryptophan, *p* < 0.001; Figures 3B,C and Supplementary Figure S2E). However, these differences did not meet significant criteria of linear mixed effect modeling (relative_abundance~host_age*exposure_type, TβMCA, *p* = 0.053; UDCA, *p* = 0.371; and tryptophan, *p* = 0.446). Overall, the gut luminal environment appeared to be pro-inflammatory and pro-atherosclerotic under IH condition.

#### IC Vs. Air

Compared to Air-controls, IC-conditioned mice also had significant differences in the beta diversity distances of both the microbiome (Figure 2A; Supplementary Figures S1A, S3E,G; pseudo-F 11.274; *p* < 0.001) and metabolome (Figure 3A; Supplementary Figures S2A, S3F,H; pseudo-F 3.046; *p* = 0.004). The magnitude of the pseudo-*F* value associated with the comparison of the IC microbiome to controls was substantially less than seen for IH, suggesting that the differences compared to the control group were not as robust. Also, the separation between IC and control samples was less obvious by the end of the study for both the microbiome and metabolome than it was for IH.

Three of the six microbial families were significantly different by linear mixed effect modeling in IC-conditioned mice as compared to controls, i.e., Peptostreptococcaceae (*p* = 0.039; Figure 2B), Ruminococcaceae (*p* = 0.003; Figure 2C), and Erysipelotrichaceae (*p* = 0.04; Figure 2D). In addition, four metabolites demonstrated overall significant differences. The relative abundances were increased for pro-atherosclerotic TβMCA (*p* = 0.013; Figure 3B), LysoPE(18:0; *p* = 0.041; Figure 3D), Lyso-PAF C-16 (*p* = 0.002; Supplementary Figure S2C), and anti-atherogenic UDCA (*p* = 0.022; Figure 3C) in IC-conditioned mice as compared to controls. While increased serum levels of acylcarnitines have been associated with adverse cardiovascular events (Strand et al., 2017), the observed changes of oleoyl L-carnitine (*p* = 0.147; Supplementary Figure S2D) under IC were less impressive than those seen in IH exposure compared to their respective controls. Collectively, IC showed the unique microbiome and metabolomic signatures that distinguish from those changes in Air and IH.

#### IH Vs. IC

Comparison of IC with IH revealed potential factors that may contribute to their different atherosclerotic phenotypes. This comparison showed significant differences in beta diversity distances of both the microbiome (Figure 2A; Supplementary Figures S1A, S3I,K; pseudo-F 50.286; *p* < 0.001) and metabolome (Figure 3A; Supplementary Figures S2A, S3J,L; pseudo-F 6.411; *p* = 0.002). The microbiome and metabolome pseudo-*F* values indicate that IH was as different from IC as it was from Air controls.

The relative abundances of the top six microbial families were significantly different between the IH and IC-conditioned mice. Similar to the differences between IH and Air, IH-conditioned mice had relatively decreased levels of Peptostreptococcaceae (*p* < 0.001; Figure 2B), Erysipelotrichaceae (*p* < 0.001; Figure 2D), and Verrucomicrobiaceae (*p* < 0.001; Supplementary Figure S1D) compared to IC-conditioned mice. Additionally, Coriobacteriaceae (*p* < 0.001; Supplementary Figure S1C) and Lachnospiraceae (*p* < 0.001; Supplementary Figure S1E) were increased in IH-conditioned mice compared to IC-conditioned mice. However, the relative abundance of Ruminococcaceae was increased under IH compared to Air controls but was decreased under IH compared to IC (*p* < 0.001; Figure 2C).

Furthermore, the difference seen in LysoPE (18:0; Figure 3D) when comparing IH to IC were similar to the difference seen in the comparison between IH and air. However, unlike the comparison between IH and Air, there was no significant difference for Lyso-PAF C-16 (*p* = 0.502; Supplementary Figure S2C) and oleoyl L-carnitine (*p* = 0.088; Supplementary Figure S2D) between IH and IC.

Together, these findings demonstrated that though IC-conditioned mice present unique differences, IH-conditioned mice differed from controls far more than IC-conditioned mice (IH > IC > Air).

## Discussion

Although OSA patients suffer from both episodic hypoxia and hypercapnia, only the impact of IH has been extensively studied from a cardiovascular viewpoint. However, IC is also a key player in the pathophysiology of human diseases (Wang et al., 2016). In this work, the respective roles of IH and IC in the progression of atherosclerosis were defined by examining them separately. We have made several important observations.

First, as compared to Air, both IH and IC accelerated the development of atherosclerosis, in an animal model of atherosclerosis. The main difference between each condition was the extent of atherosclerosis in the PA vs. the aorta/aortic arch. More importantly, this study convincingly showed that IC alone can promote atherosclerosis.

Second, IH exacerbated atherosclerosis in the PA far more than it did in the aorta. One possible explanation for this is the inherent response of the vascular bed to hypoxia. Depending on its extent and duration, hypoxia can cause vasodilation in most tissues of the body. By contrast, in the lungs, hypoxia induces vasoconstriction resulting in pulmonary arterial hypertension and damage to endothelial cells, both of which are atherogenic. Another possibility is the vascular response to the microbiome or the metabolome that results from the interaction between the host and gut microbiota. This is a still a major area of investigation, and it is difficult at present to dissect the effect of the microbiota, inflammatory response, and cytokines on the blood vessels themselves.

We have shown that atherosclerosis can be promoted by IHC in the PA trunk and its proximal branches of both *ApoE^−/−^* and *Ldlr^−/−^* mice (Douglas et al., 2013; Xue et al., 2017; Imamura et al., 2019), demonstrating that the effect of IHC on the PA is not genetic background-dependent. The current IH and IC data corroborate the notion that PA atherosclerosis is promoted by these blood gas changes as well. Of note, our previous published data show that right ventricular maximum pressure and isovolumic relaxation were significantly increased and left ventricular fractional shortening was reduced with IHC, suggestive of pulmonary hypertension (PH), and left ventricular dysfunction (Douglas et al., 2013). The causal relationship between PA atherosclerosis and PH is not clear. PA atherosclerosis has been observed in patients with diseases that cause PH, such as atrial septal defect and shunt and chronic obstructive pulmonary disease (Russo et al., 1999; Nascimento et al., 2009), and was associated with hypertensive pulmonary vascular disease (Moore et al., 1982). As a matter of fact, OSA patients have elevated PA pressure (Minai et al., 2009) and a higher incidence of acute pulmonary embolism (Alonso-Fernandez et al., 2013).

In the past, greater attention was given to OSA-related hypoxia. IH alone with HFD exacerbated aortic atherosclerosis in *ApoE^−/−^* mice (Jun et al., 2010). However, IH did not seem to increase aortic lesions compared to Air controls in our study. This discrepancy between previous studies and ours is probably related to multiple factors including the specific experimental protocol of IH exposure, exposure duration, and composition of HFD.

Third, current study demonstrated that IC alone facilitates the development of atherosclerosis in the aorta and PA. Previous studies have shown that hypercapnia has both beneficial and deleterious effects (Shigemura et al., 2017). On the one hand, hypercapnia has been associated with improved outcome in patients with acute lung injury (Acute Respiratory Distress Syndrome Network et al., 2000), which is thought to be mediated by inhibition of the NF-κB inflammatory pathway (Contreras et al., 2015). In addition, hypercapnia inhibits hypoxia-induced pulmonary vascular remodeling (Ooi et al., 2000) and prevents hypoxia-induced PH (Kantores et al., 2006). On the other hand, hypercapnia injures alveolar epithelial cells (Lang et al., 2000), impairs lung edema clearance (Vadasz et al., 2008), and reduces alveolar epithelial repair (O’Toole et al., 2009). It also modulates innate immunity and host defense that increase the susceptibility to and mortality of pulmonary infections (Gates et al., 2013).

It is known that the gut microbiota might affect atherogenesis *via* modulation of cholesterol and lipids metabolism as well as host immune response (Chistiakov et al., 2015; Jonsson and Backhed, 2017). Our previous study has demonstrated that 8-week IHC, in spite of more atherosclerosis, did not cause additional increase in total plasma cholesterol or triglyceride levels as compared to Air controls, both fed with HFD, in *Ldlr^−/−^* mice (Douglas et al., 2013). However, IH alone was reported to cause hyperlipidemia in *ApoE^−/−^* mice (Savransky et al., 2007; Jun et al., 2010). Intriguingly, we found that mice exposed to IHC, IH, and IC, even with reduced body weights (IHC and IH) or no weight change (IC; data not shown), developed more atherosclerotic lesions than Air control mice. Collectively, these findings imply that IH and IC, either singularly or in combination, may accelerate atherosclerotic formation *via* mechanisms other than hyperlipidemia. To corroborate this notion, a recent finding (Kasahara et al., 2017) showed that germ-free *ApoE^−/−^* mice had reduced atherosclerotic lesions despite hypercholesterolemia when compared with conventionally-reared mice. This decrease in atherosclerosis was associated with the attenuation of lipopolysaccharides (LPS)-mediated inflammatory responses. Commensal bacteria, such as *Firmicutes* sp. and *Bacteroidetes* sp., could release LPS, that work *via* Toll-like receptor (TLR) 4 signaling to elevate lipid uptake by macrophages and induce their transformation to foam cells, a hallmark of early atherosclerosis (Chistiakov et al., 2015). Moreover, LPS damage gut epithelial integrity and enhance systemic inflammation, which in turn promote atherosclerosis (Chistiakov et al., 2015). In addition to LPS, microbiota can also regulate the levels of inflammatory cytokines, such as interleukin-6 (IL-6), Interleukin-8 (IL-8), and tumor necrosis factor *α* (TNF-α), thereby indirectly affecting atherosclerosis progression (Chistiakov et al., 2015). Conversely, probiotics have athero-protective effects *via* down-regulating inflammation or ameliorating dyslipidemia (Chistiakov et al., 2015). Our current study has identified microbiota and metabolites that were altered by IH/IC and were shown to be associated with inflammation and lipid metabolism by other studies, suggesting that these microbiota and metabolites may contribute to atherosclerosis *via* regulating inflammation and lipid metabolism.

Gut microbiota can have either protective or deleterious effects in the host. In terms of atherosclerosis, bacterial family Peptostreptococcaceae has been shown to be positively associated with both an omnivorous diet and trimethylamine N-oxide (TMAO) production in humans (Koeth et al., 2013), suggesting that its potential role in the metabolism of dietary L-carnitine into trimethylamine (TMA). TMA is further oxidized to TMAO by hepatic flavin monooxygenases. TMAO enhances macrophage cholesterol accumulation, foam cell formation, and atherosclerosis, and all correlated with an increased risk of heart attack, stroke, and death (Wang et al., 2011; Tang et al., 2013). Increased relative abundance of Peptostreptococcaceae was observed after 4-week IH and IC exposures, 2 weeks before the atherosclerosis became evident, indicating that Peptostreptococcaceae plays a role in IH- and IC-induced atherosclerosis.

*Akkermansia muciniphila*, the only genus of family Verrucomicrobiaceae in mammals, is believed to have health benefits in humans. Its abundance is known to be inversely correlated with obesity, diabetes, and cardiometabolic diseases including atherosclerosis and low-grade inflammation (Cani and de Vos, 2017). The relative abundance of *A. muciniphila* was ultimately decreased under IH and presumed to have been more permissive to the development of atherosclerotic lesions. Based on recent findings (Li et al., 2016a), reduced *A. muciniphila* under IH may contribute to IH-induced atherosclerosis by causing a leaky gut, which allows pro-inflammatory mediators to be more readily absorbed systemically. However, increased abundance of *A. muciniphila* was found under IC, potentially indicating an attempt to return to homeostasis.

Another interesting example linking atherosclerosis to microbiota is related to the bacterial families Ruminococcaceae, Lachnospiraceae, and Coriobacteriaceae. Previous studies have shown that Ruminococcaceae and Lachnospiraceae were positively correlated with atherosclerotic lesion size in *ApoE^−/−^* mice (Wang et al., 2015; Liu et al., 2018), and Coriobacteriaceae was enriched in patients with symptomatic atherosclerosis (Karlsson et al., 2012). These findings suggest that these bacteria play an important role in atherogenesis in our mice. Although the underlying mechanism is not well understood, it is often attributed to their roles in inflammatory pathways (Martinez-Medina et al., 2006; Andoh et al., 2007), lipid metabolism (Martinez et al., 2009; Liu et al., 2015), and TMAO production (Wang et al., 2015; Qiu et al., 2018; Chen et al., 2019). Ruminococcaceae and Lachnospiraceae species hydrolyze starch and other sugars to produce butyrate and other short-chain fatty acids (SCFAs). SCFAs are ligands for the G protein-coupled receptors 41 and 43 and are hypothesized to reduce inflammatory pathways by activating these receptors (Biddle et al., 2013; Ohira et al., 2017). Notably, Ruminococcaceae and Lachnospiraceae contain bile acid inducible (*bai*) genes, which encode enzymes involved in 7α-dehydroxylation and can convert host primary bile acids to secondary bile acids (Vital et al., 2019). The secondary bile acids act through farnesoid X receptor (FXR) and G-protein-coupled bile acid receptor (TGR5) and affect host metabolism and health. In fact, both FXR and TGR5 have been implicated in atherosclerosis (Hanniman et al., 2005; Miyazaki-Anzai et al., 2014, 2018).

One of the top differentially abundant metabolites is TβMCA, which is a primary bile acid synthesized in mice. Evidence has shown that the level of TβMCA can be affected by gut microbiota (Sayin et al., 2013) and induced by TMAO, which is an important molecule in the development of atherosclerosis (Ding et al., 2018). Since (a) TβMCA is a naturally occurring FXR antagonist (Sayin et al., 2013) and (b) it has been reported that loss of functional FXR increases atherosclerotic lesions in *ApoE^−/−^* mice along with a more atherogenic plasma lipid and lipoprotein profile (Hanniman et al., 2005), it is likely that the elevated TβMCA under IH and IC facilitates atherosclerotic formation.

Increased abundance was detected for Lyso-PAF C-16 under IH and IC conditions. Lysophosphatidylcholine is known to promote atherosclerosis by various mechanisms including activating endothelial cells to produce reactive oxygen species, enhancing the release of pro-inflammatory cytokines, attracting immune cells to the vascular endothelial wall, and mediating atherogenic activity of ox-LDL (Androulakis et al., 2005; Matsumoto et al., 2007; Li et al., 2016b). This suggests that Lyso-PAF C-16 plays an important role in the progression of atherosclerosis induced by IH and IC.

## Conclusion

Taken altogether, we are the first to show that IC, in addition to IH, contributes to OSA-related atherosclerosis. Moreover, we also demonstrate that IHC, a hallmark of OSA, change the gut microbiota and metabolites. The changes in the gut luminal environment likely influence the development of atherosclerosis by modulating host gut permeability, inflammatory responses, microbial metabolites TMA/TMAO, and bile acid and lipid metabolism. The knowledge obtained in the current study paves the way for a better understanding of the mechanistic link between IH/IC, gut microbiome, and OSA-induced atherosclerosis.

## Data Availability Statement

The datasets presented in this study can be found in online repositories. The names of the repository/repositories and accession number(s) can be found in the article/Supplementary Material.

## Ethics Statement

The animal study was reviewed and approved by the Animal Care Committee of the University of California, San Diego.

## Author Contributions

GH, JX, DZ, AZ, PD, and RK: conception or design. JX and OP: acquisition of data. JX, CA, DZ, OP, CM, LJ, AT, and EE: analysis and interpretation of data. JX and CA: drafting the work. JX, CA, DZ, OP, CM, LJ, AT, EE, PD, RK, AZ, and GH: revision and final approval of the manuscript. All authors contributed to the article and approved the submitted version.

### Conflict of Interest

AZ is a co-founder and equity holder of Tortuga Biosciences.

The remaining authors declare that the research was conducted in the absence of any commercial or financial relationships that could be construed as a potential conflict of interest.

## Footnotes

^1^http://www.earthmicrobiome.org/protocols-and-standards/16s

^2^http://biocore.github.io/calour/index.html#

^3^https://github.com/knightlab-analyses/longitudinal-osa

^4^https://gnps.ucsd.edu/ProteoSAFe/static/gnps-splash.jsp

^5^https://github.com/knightlab-analyses/longitudinal-osa

## Abbreviations

OSA: Obstructive sleep apnea
IHC: Intermittent hypoxia and hypercapnia
IH: Intermittent hypoxia
IC: Intermittent hypercapnia
ApoE: Apolipoprotein E
Ldlr: Low density lipoprotein receptor
HFD: High-fat diet
PA: Pulmonary artery
PH: Pulmonary hypertension
ASVs: Assigned sequence variants
LC–MS/MS: Liquid chromatography-tandem mass spectrometry
mzXML: *m/z* extensible markup language
PCoA: Principal coordinates analysis
LysoPE(18:0): 1-Stearoyl-2-hydroxy-sn-glycero-3-phosphoethanolamine
Lyso-PAF C-16: 1-Hexadecyl-sn-glycero-3-phosphocholine
UDCA: Ursodeoxycholic acid
TβMCA: Tauro-β-muricholic acid
TMAO: Trimethylamine N-oxide
FXR: Farnesoid X receptor
TGR5: G-protein-coupled bile acid receptor, Gpbar1
