# Supplementary material for: Influence of Intermittent Hypoxia/Hypercapnia on Atherosclerosis, Gut Microbiome, and Metabolome
Source: Front Physiol. 2021 Apr 8;12:663950. doi: 10.3389/fphys.2021.663950 (PMC8060652; doi:10.3389/fphys.2021.663950)
Supplement: Supplementary file 1 [file Data_Sheet_1.PDF]

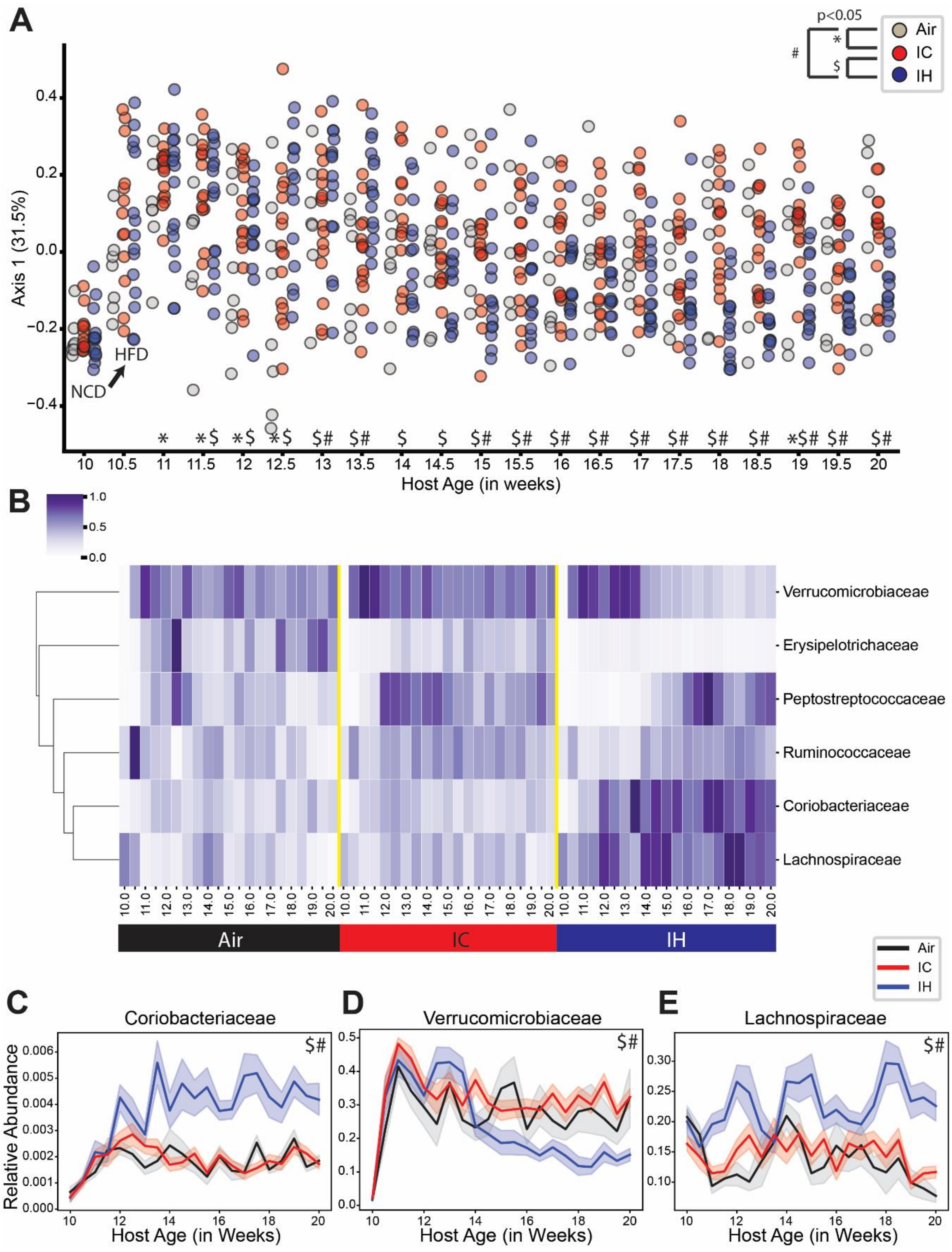

Figure S1. Additional 16S microbiome of *ApoE*$^{-/-}$ mice on HFD during chronic 10-wk treatment. **(A)** Axis 1 of the Weighted UniFrac PCoA of the microbiome for all measured time points. **(B)** Relative abundance heatmap (row normalized, set on standard scale) compared by exposure types over time. Longitudinal relative abundance values for: **(C)** family Coriobacteriaceae; **(D)** family Verrucomicrobiaceae. The only ASV in this family belongs to *Akkermansia muciniphila;* **(E)** family Lachnospiraceae. PERMANOVA used for statistical comparisons at the population level. Linear Mixed Effect (LME) modeling used for statistical comparisons of individual microbiota. The shaded areas in parts **C-E** represent standard error of the mean. Air/controls are black (n=6), IH is blue (n=12), IC is red (n=12). Statistical significance $p<0.05$, * IC vs Air, $^{\#}$ IH vs Air and $^{\$}$ IH vs IC.

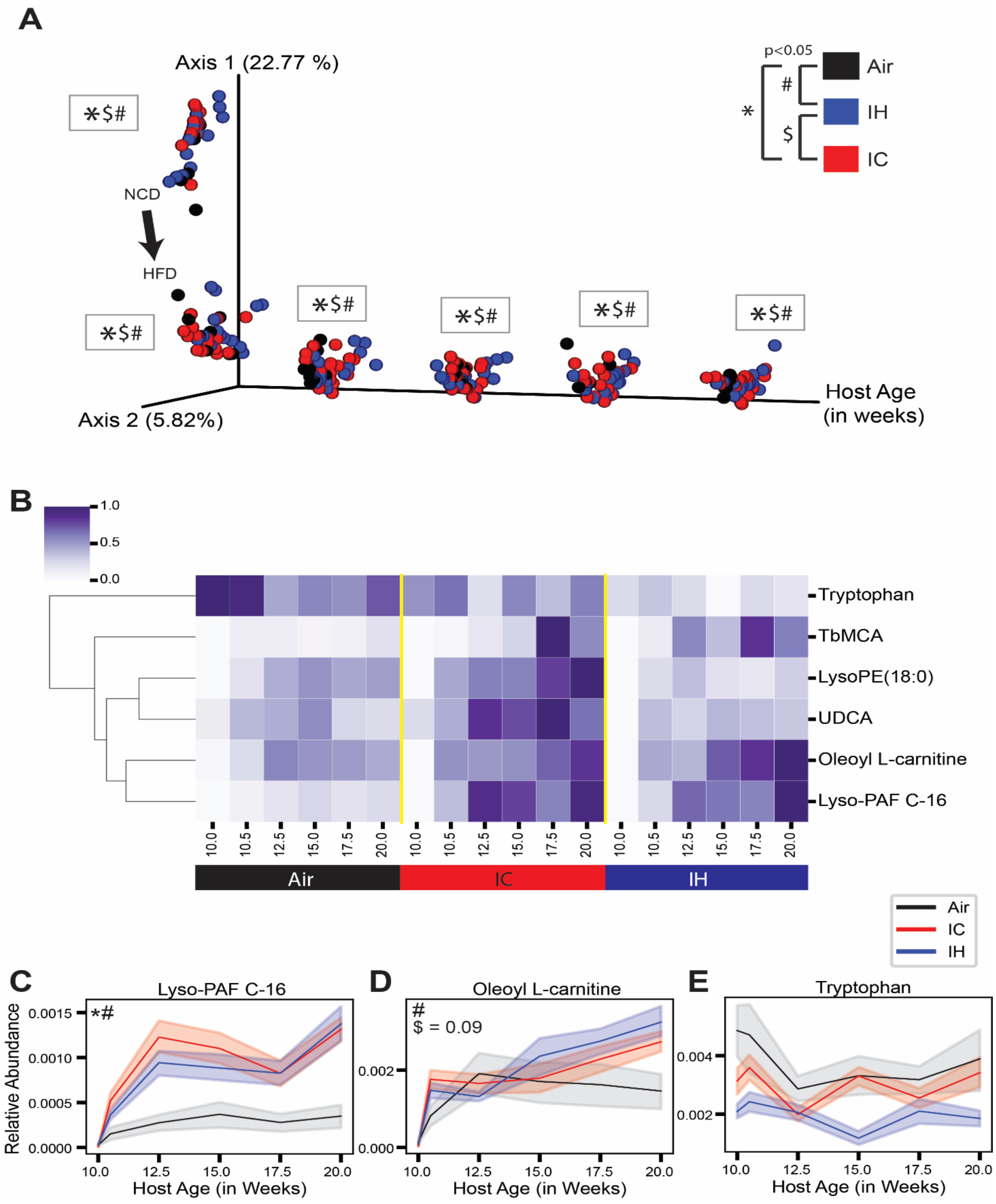

Figure S2. Additional Untargeted LC-MS/MS metabolomics of *ApoE*$^{-/-}$ mice on HFD during chronic 10-wk treatment. (A) Canberra PCoA of the metabolome for all measured time points. **(B)** Relative abundance heatmap (row normalized, set on standard scale) compared by exposure types over time. Longitudinal relative abundance values for: **(C)** lysophosphocholine, 1-Hexadecyl-sn-glycero-3-phosphocholine [Lyso-PAF C-16] [Level 3 identification]; **(D)** acylcarnitine, oleoyl L-carnitine [Level 3 identification]; **(E)** amino acid, tryptophan [Level 3 identification]. PERMANOVA used for statistical comparisons at the population level. Linear Mixed Effect (LME) modeling used for statistical comparisons of individual metabolites. The shaded areas in parts **C-E** represent standard error of the mean. Air/controls are black (n=6), IH is blue (n=12), IC is red (n=12). Statistical significance $p<0.05$, * IC vs Air, $^{\#}$ IH vs Air and $^{\$}$ IH vs IC.

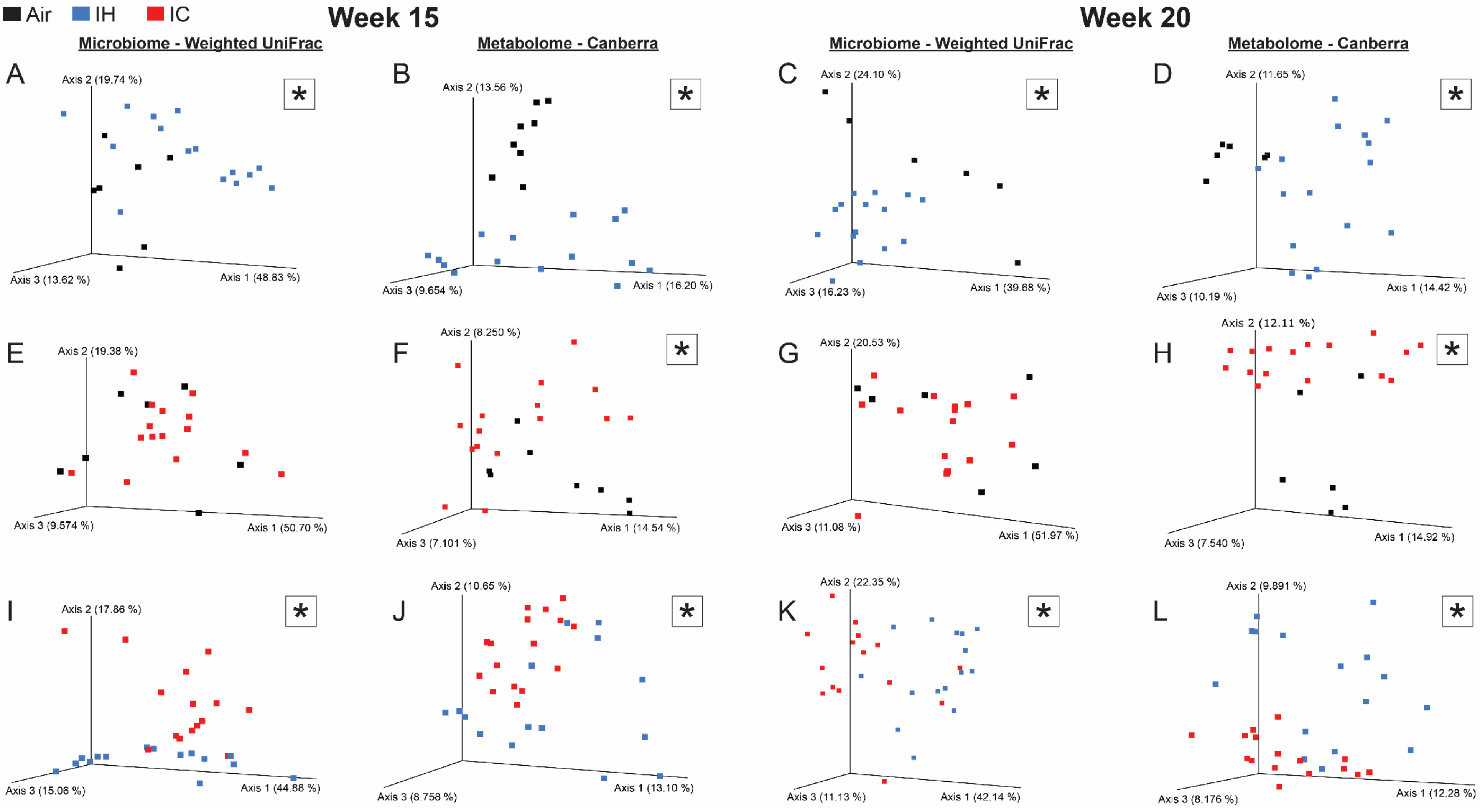

Figure S3 Additional Microbiome and Metabolome PCoA for Weeks 15 and 20. (A) Microbiome Weighted UniFrac PCoA Week 15 Air vs IH only. **(B)** Metabolome Canberra PCoA Week 15 Air vs IH only. **(C)** Microbiome Weighted UniFrac PCoA Week 20 Air vs IH only. **(D)** Metabolome Canberra PCoA Week 20 Air vs IH only. **(E)** Microbiome Weighted UniFrac PCoA Week 15 Air vs IC only. **(F)** Metabolome Canberra PCoA Week 15 Air vs IC only. **(G)** Microbiome Weighted UniFrac PCoA Week 20 Air vs IC only. **(H)** Metabolome Canberra PCoA Week 20 Air vs IC only. **(I)** Microbiome Weighted UniFrac PCoA Week 15 IC vs IH only. **(J)** Metabolome Canberra PCoA Week 15 IC vs IH only. **(K)** Microbiome Weighted UniFrac PCoA Week 20 IC vs IH only. **(L)** Metabolome Canberra PCoA Week 20 IC vs IH only. Air/controls are black (n=6), IH is blue (n=12), IC is red (n=12). Statistical significance * $p<0.05$ by PERMANOVA.
